# Hazard analysis and critical control points for foods consumed by children aged 6–24 months in Maputo, Mozambique

**DOI:** 10.1371/journal.pgph.0005497

**Published:** 2026-07-07

**Authors:** Álvaro Manhiça, Clara MacLeod, Mahira Amade, Joelma Baduro, Miguel Guambe, Yolanda Manganhe, Idalécia Cossa-Moiane, Igor Ubisse Capitine, Jackie Knee, Julie Watson, Edna Viegas, Oliver Cumming

**Affiliations:** 1 Instituto Nacional de Saúde (INS), Maputo, Mozambique; 2 Department of Disease Control, London School of Hygiene and Tropical Medicine, London, United Kingdom; North Carolina State University, UNITED STATES OF AMERICA

## Abstract

Diarrhoeal disease remains a significant cause of mortality among children under five, particularly in high-burden settings where food consumed by young children is frequently contaminated. Understanding food preparation, feeding, and storage practices for children aged 6–24 months is essential to inform interventions that reduce diarrhoeal disease. Using the Hazard Analysis and Critical Control Points (HACCP) approach, we identified critical control points (CCPs) associated with potential food contamination among households with a child aged 6–24 months in the Polana Caniço neighbourhood of Maputo, Mozambique. We conducted 110 structured observations of household food hygiene practices, including food handling, preparation, cooking, feeding, and storage, to identify key hazards and CCPs for microbial contamination of foods consumed by young children. Food flow diagrams were developed for the main food groups to support the HACCP analysis. Our systematic assessment of food hygiene behaviours identified several key practices to target: cleaning utensils used for child feeding, caregiver handwashing with soap before feeding, boiling liquids added to food, storing cooked food in clean containers, and thoroughly cooking and reheating food before feeding. While these CCPs are consistent with those identified in similar settings, our findings provide context-specific evidence on food hygiene practices in an urban informal setting. These findings can inform future research to evaluate the relative importance of identified hazards and CCPs and guide policymakers and practitioners in designing household- and community-level interventions to reduce foodborne health risks among young children.

## Introduction

Foodborne disease, caused by the consumption of food contaminated with bacteria, viruses, parasites, or chemicals, is a major public health concern. The World Health Organization (WHO) estimates that 33 million disability-adjusted life years (DALYs) are attributable to the consumption of contaminated food globally [1]. However, the burden of foodborne disease is highest in low- and middle-income countries, particularly in sub-regions of Africa, where nearly 70% of the burden is due to diarrhoeal disease agents, such as Campylobacter [1]. This also disproportionately affects children under the age of five, with an estimated 40% of the global foodborne disease burden borne by this age group [1].

Diarrhoea, a common symptom of foodborne disease, is a leading cause of morbidity and mortality among children under five years of age. Globally, most diarrhoeal deaths occur in children under the age of two, with over 50% occurring in children between 6 and 11 months [2]. Children typically transition from exclusive breastfeeding to consuming complementary food between the ages of 6–24 months old. The risk of diarrhoeal disease increases during this period due to a simultaneous decrease in immune protection from reduced breastmilk and increase in exposure to potentially contaminated food [3].

Most food contamination and recurrent foodborne infections originate at the household level, primarily due to exposure to faecal pathogens in the domestic environment [4]. For children in the complementary feeding period, food safety is largely influenced by caregivers, who are responsible for the preparation, cooking, feeding, and storage of the child’s food [5,6]. Risk factors for the microbial contamination of food include poor handwashing practices, cross-contamination between raw and cooked foods, inadequate food storage (i.e., storing cooked or perishable foods at unsafe temperatures or in unclean or non-sealed containers that allow microbial growth or recontamination), insufficient cooking or reheating, and unclean cooking or eating utensils [7–10]. Understanding caregiver food hygiene practices in high-burden settings can inform targeted interventions to prevent foodborne diseases in young children [11].

The Hazard Analysis and Critical Control Points (HACCP) approach is a method recommended by the World Health Organization (WHO) to identify the causes of food-associated hazards in domestic settings and to determine preventive measures [12]. The approach involves several steps, including identifying hazards associated with food contamination and then determining critical control points that can be applied to reduce or control those hazards. Previous studies found an association between food hygiene practices in the household and the risk of food contamination using the HACCP approach [7,10]. For example, Wells et al. (2024) found that children feeding themselves and exposure to animals were risk factors for food contamination among children discharged from acute malnutrition programmes in South Sudan [10]. Meanwhile, Bick et al. (2020) identified handwashing with soap, cooking, and storage practices as risk factors for food contamination among households enrolled in a sanitation intervention trial in Maputo, Mozambique [7,10]. Studies in Bangladesh and Mali also adopted the HACCP approach to identify key points of potential food contamination and implemented a health education intervention based on the identified critical points [8,13]. Other studies in Nepal and Mali applied the HACCP approach and a behaviour change strategy to improve food hygiene practices [14,15]. The identification of critical control points via the HACCP approach can be useful for targeting resources and developing control strategies to reduce exposure to food contamination, especially among young children in high-burden settings.

### Aim and objectives

The aim of the study was to identify practices that may contribute to the contamination of foods consumed by children aged 6–24 months in the Polana Caniço neighbourhood of Maputo, Mozambique. The objectives were to: 1) characterise behaviours related to young children’s food, including preparation, cooking, feeding, storage, and reheating, 2) identify critical control points based on observed practices, and 3) develop food flow diagrams of the preparation, storage, and feeding of the main foods consumed by children aged 6–24 months.

## Methods

### Ethical statement

Ethical approval was obtained from the London School of Hygiene and Tropical Medicine, Research Ethics Committee, London, UK (Ref: 17188) and National Committee on Bioethics for Health (CNBS) Comité Nacional de Bioética para a Saúde (CNBS) (Ref: 591/CNBS/19) in Mozambique. All study participants provided written informed consent (for both their and their child’s participation). Additional information regarding the ethical, cultural, and scientific considerations specific to inclusivity in global research is included in the supporting information (S1 Checklist).

### Study design and setting

This is a cross-sectional HACCP study that was conducted as part of the Urban Infant Foodscape (UIF) study implemented in Kenya and Mozambique. The aim of UIF was to estimate the burden of foodborne disease among children aged 6–24 months in low-income, high-density urban areas of Nairobi, Kenya, and Maputo, Mozambique.

**Fig 1:**
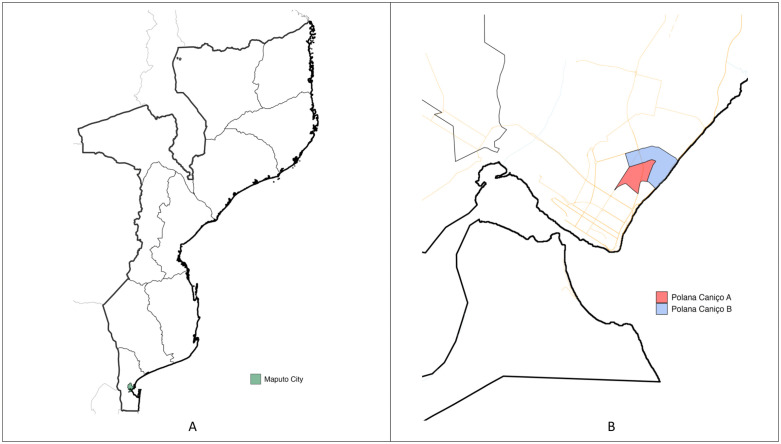
Map of Polana Caniço A and B, Maputo, Mozambique. A. Map of Maputo City, Mozambique. B. Map of the Polana Caniço A and Polana Caniço B neighbourhoods within Maputo City. The base layer for both maps was obtained from the Database of Global Administrative Areas (GADM) (available from: https://gadm.org/download_country.html; terms of use: https://gadm.org/license.html). Road and river features were obtained from OpenStreetMap under the Open Database License (available from: https://www.openstreetmap.org). Neighbourhood boundaries for Polana Caniço A (relation ID: 11303649) and Polana Caniço B (relation ID: 11303648) were also obtained from OpenStreetMap.

This study was undertaken in Polana Caniço, a peri-urban neighbourhood located in Maputo, Mozambique (Fig 1A and Fig 1B), from 12/07/2022–22/08/2023. The Polana Caniço neighbourhood is in the KaMaxaqueni District, the third most populated district of Maputo, with approximately 204,000 inhabitants [16]. The total area of Polana Caniço is estimated to be 678.6 hectares. The area is divided into two areas: Polana Caniço A (212.3 hectares) and Polana Caniço B (466.3 hectares). Polana-Caniço is composed of 125 blocks (77 and 48 in Polana Caniço A and B, respectively) and 17,228 households (8,464 and 8,764 in Polana Caniço A and B, respectively) [16]. The study was implemented by the Polana Caniço Health Research and Training Centre (CISPOC) at the National Institute of Health, Mozambique.

### Eligibility and enrolment

The sampling frame was drawn from the UIF participant list. The UIF study enrolled 526 households in the Polana Caniço neighbourhood of Maputo, Mozambique, each with at least one child aged 6–24 months at the time of the study. A subset of 110 households was randomly selected for household observations. Households were randomly selected from the sampling frame by the CISPOC data manager using Stata Version 18 (Stata Corp, College Station, TX, USA). Households were eligible to participate in the structured observations if both the child and their caregiver were present and available at the time of observation. Any other adult in the household over the age of 18 involved in food preparation, feeding, and storage was also eligible to participate, provided they gave written informed consent.

### Data collection: household survey and structured observations

Information on household characteristics, water, sanitation, and hygiene (WASH) conditions, and food preparation, feeding, and storage practices was drawn from the household survey administered to the 526 households in the UIF study. After consenting to additional structured observations, enumerators recorded the type of food served to the child; preparation and storage of ingredients; separation of cooked and raw foods; food preparation and feeding surfaces; handwashing before preparation and feeding; utensils used; food additions before and after cooking; reheating practices; feeding practices; food storage areas; and animal contact during food preparation and feeding. Foods included in the structured observation were required to have been prepared within the preceding 24 hours, although the exact duration of storage prior to observation was not measured. During these observations, enumerators also recorded the presence of a handwashing station and availability of soap and water.

The structured observations were conducted from 26/04/2023–22/08/2023. They targeted specific food hygiene practices and therefore only lasted between 30 minutes and 1 hour, depending on the duration of preparation, feeding, and food storage activities in each household. Observations were recorded on tablets using forms developed in Open Data Kit (ODK). The forms were written in English and translated to Portuguese. All data were stored and encrypted on a secure server.

### HACCP methodology

**Fig 2:**
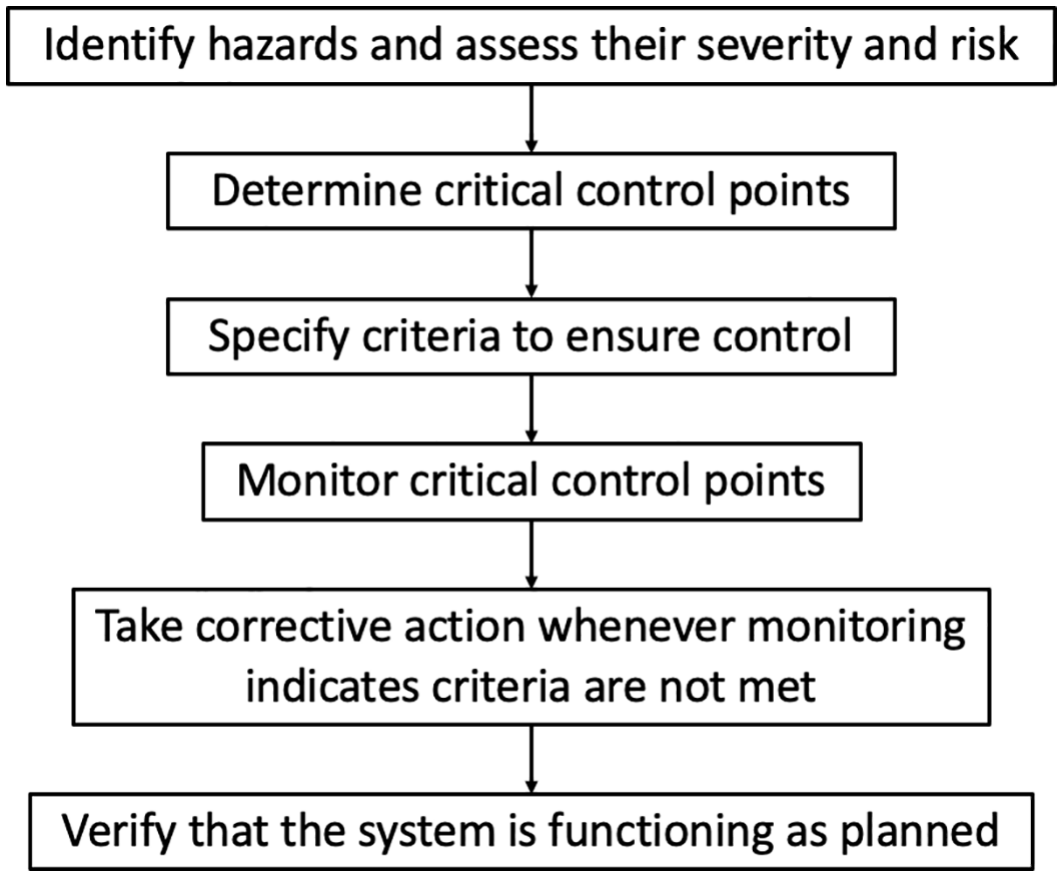
Components of the HACCP system [17].

The HACCP system is illustrated in Fig 2. This paper focuses on the first three steps: 1) identifying hazards, 2) determining critical control points, and 3) specifying control measures. Structured observations of household food hygiene practices were used to identify key hazards and critical control points (CCPs) for microbial contamination of foods consumed by children. A CCP is a point in the process at which action is required to eliminate or reduce hazards associated with pathogenic bacteria. To determine whether a step or procedure was a CCP, we assessed whether control or corrective measures could be applied to prevent or minimise the hazard. For example, food storage is a CCP if storage conditions (time and temperature) are not controlled and food is served without reheating. Data from observations were also used to develop food flow diagrams describing food preparation, feeding, and storage practices for the different types of foods consumed by children enrolled in the study.

### Data analysis

Variables from the observations were converted to binary or categorical variables, with food types grouped based on common ingredients and food preparation processes. Households were categorised according to the WHO/UNICEF Joint Monitoring Programme (JMP) ladder definitions for drinking water, sanitation, and hygiene [18]. We calculated frequencies of key food hygiene behaviours. Data were analysed in Stata Version 18 (Stata Corp, College Station, Texas, USA).

## Results

### Characteristics of study participants

A total of 110 households were included in the analysis. Caregivers responsible for child feeding were predominantly female (96%) and often the child’s mother (87%). The mean caregiver age was 30 years (range 18–64 years), and most caregivers had completed primary or secondary education (88%). The mean age of the child was 19 months (range 9–24 months). The median number of household members was 5 (interquartile range [IQR] 4–6), and most households had only one child under the age of five (IQR 1–2). Household floors were predominantly made of cement (86%) and walls of cement blocks or bricks (99%). The majority of households had electricity (95%). The largest proportion of households (35%) reported a monthly income between 5,000 and 9,999 Mozambican meticals, equivalent to approximately 80–157 United States dollars.

### Water, sanitation, and hygiene

Most households had access to a basic drinking water service (87%) (i.e., using an improved source with a round-trip collection time of no more than 30 minutes, including queueing). Only 10% of households reported treating their water before consumption. Of those that did, most reported boiling water. Ninety-seven percent of households stored their water in a container with a closed lid. Households commonly collected 200–250 litres of water per day (34%), with half of households (50%) collecting enough water to meet the World Health Organization minimum water requirement of 50 litres per person per day [19].

Most households had access to limited sanitation services (i.e., an improved facility shared between two or more households) (41%) or safely managed sanitation services (31%). A quarter of households had access to basic sanitation services (i.e., an improved facility not shared with other households) (25%). Almost half of all households did not have an observed handwashing facility (44%). The remaining households had access to either a limited handwashing facility (25%) (i.e., a handwashing facility lacking soap and/or water) or a basic handwashing facility (32%) (i.e., a handwashing facility with soap and water at home). Soap was present in 35% of households, but only 32% of households had access to both soap and water for handwashing.

Garbage in the compound was observed in 24% of households. Seventy-six percent of households had signs of rodent presence, and animal faeces were observed in 7% of households. At the time of the household survey, most caregivers reported washing their hands with soap and water before food preparation (68%), child feeding (66%), and after using the toilet (82%). However, only 39% of caregivers reported washing their hands after changing the child’s diaper (Table 1).

**Table 1:** Characteristics of the 110 households included in the study.

Variable	Description	n (N= 110)	(%)
**Household characteristics**			
Infant age (months)	6 – 12	7	(6%)
	12 – 18	47	(43%)
	18 – 24	56	(51%)
Caregiver gender	Female	106	(96%)
Caregiver’s relationship to infant	Mother	96	(87%)
	Grandmother	6	(6%)
	Father	5	(6%)
	Aunt	2	(2%)
	Sister	1	(1%)
Caregiver age	18 – 30	70	(64%)
	31 – 45	31	(28%)
	46 – 60	7	(6%)
	61+	2	(2%)
Level of education completed by caregiver	None	7	(6%)
	Primary	34	(31%)
	Secondary	63	(57%)
	University	5	(5%)
	Do not know	1	(1%)
Household size	Median (IQR)	5	(4–6)
Households with children under five	Median (IQR)	1	(1–2)
Monthly household income	<2,500	9	(8%)
	2,500 – 4,999	28	(25%)
	5,000 – 9,999	39	(35%)
	10,000 – 14,999	13	(12%)
	15,000 – 19,999	2	(2%)
	Don’t know	12	(11%)
	Prefer not to say	7	(6%)
**Water, sanitation, and hygiene (WASH) conditions**			
Drinking water source (JMP)	Safely managed	3	(3%)
	Basic	96	(87%)
	Limited	11	(10%)
Drinking water storage (observed)	Closed container	108	(98%)
	Open container	2	(2%)
Water treatment (reported)	Yes	11	(10%)
Sufficient volume of water collected per day (reported)	Yes	55	(50%)
Sanitation level (JMP)	Safely managed	34	(31%)
	Basic	27	(25%)
	Limited	45	(41%)
	Unimproved	4	(4%)
Handwashing facility available (observed)	Yes	62	(56%)
Observed soap and water available at the handwashing facility (observed), N = 62	Yes	35	(56%)
Handwashing with soap and water in the past 24 hours (reported)	Before food preparation	75	(68%)
	Before feeding	73	(66%)
	After diaper change	43	(39%)
	After using the toilet	91	(82%)
Presence of garbage or waste in compound	Yes	26	(24%)

### Structured observations and HACCP approach

The frequencies of observed preparation, cooking, feeding, and storage practices for foods consumed by children are presented by food type (Table 2). Foods were grouped into eight categories: cereals (n = 60), cereals with vegetables (n = 14), cereals with meat or fish (n = 13), cereals with legumes (e.g., beans) (n = 6), fried eggs (n = 6), salads (n = 6), starchy vegetables (n = 4), and bread (n = 4). Cereal-based foods included porridge, rice, and xima. Cereals with vegetables commonly included cabbage curry and rice with tomato curry, while cereals with meat or fish typically included chicken or dried fish. Starchy vegetables included potatoes and cassava. A total of 314 individual raw ingredients were observed across households.

**Table 2:** Food preparation, cooking, feeding, and storage practices among households with children aged 6–24 months in Maputo, Mozambique. Percentages exclude observations where practices were not applicable. Denominators are provided for each variable.

Variable	Value	n	(%)
**Observed practices during food preparation**			
Primary food preparer, N = 110	Caregiver	95	(86%)
	Not caregiver	8	(7%)
	Caregiver and someone else	7	(7%)
Types of food served to child, N = 110	Cereals	60	(55%)
	Cereals with vegetables (e.g., cabbage, tomato, carrot)	14	(13%)
	Cereals with meat or fish (e.g., chicken or dried fish)	13	(13%)
	Cereals with legumes (e.g., beans)	6	(5%)
	Fried egg	6	(5%)
	Starch vegetables	4	(4%)
	Bread	4	(4%)
	Salad	3	(3%)
Timing of food preparation, N = 110	Prepared during the observation	76	(69%)
	Prepared before the observation (e.g., one day before)	34	(31%)
Source of ingredients, N = 314	Local market	158	(50%)
	Street stall	44	(14%)
	Home grown	36	(11%)
	Supermarket	33	(11%)
	Small shop	27	(9%)
	Farm	8	(3%)
	Other	6	(2%)
	Neighbour	2	(1%)
Storage location of raw ingredients, N = 314	Inside the home	281	(89%)
	Outside the home	24	(8%)
	Fridge	6	(2%)
	Freezer	3	(1%)
Storage method of ingredients, N = 284	Covered	257	(90%)
	Not covered	27	(10%)
	Ingredient not stored prior to preparation	30	–
Raw and cooked foods separated, N = 110	Yes	102	(93%)
Ingredients washed, N = 102	No	100	(98%)
	Yes	2	(2%)
	Ingredient did not require washing (e.g., dry goods, reheated food)	212	–
Liquids added during cooking, N = 86	No	48	(56%)
	Yes (e.g., water, milk, tea)	38	(44%)
	Food not freshly cooked during observation	24	–
Food preparation surface, N = 85	Table	77	(91%)
	Container	5	(6%)
	Floor with no mat	2	(2%)
	Floor with mat	1	(1%)
	Food prepared prior to observation	25	–
Food preparation location, N = 90	Inside	63	(70%)
	Outside	22	(24%)
	Equally outside and inside	5	(6%)
	Food prepared prior to observation	20	–
Caregiver handwashing with soap, N = 110	Yes	26	(24%)
Washing food preparation surface with soap and water, N = 80	Yes	2	(3%)
Cleaning utensils with soap and water, N = 316	Yes	46	(15%)
Food heated to boiling, N = 101	Yes	73	(72%)
	No	28	(28%)
	Food not heated during preparation (e.g., salad, bread)	9	–
**Observed practices before feeding the child**			
Liquids added after cooking (e.g., water, milk, tea), N = 86	No	75	(87%)
	Yes, and heated to boiling	6	(7%)
	Yes	5	(6%)
	Food not freshly cooked during observation	24	–
Pre-prepared food heated to boiling, N = 34	Yes	22	(65%)
Handwashing with soap and water, N = 110	Caregiver or other person responsible for child feeding	17	(15%)
	Child	4	(4%)
**Observed practices during child feeding**			
Who fed the child, N = 110	Caregiver	96	(87%)
	Not caregiver (e.g., sibling, aunt)	8	(7%)
	Caregiver and other	6	(6%)
Child seating location, N = 110	Caregiver’s knee/lap	62	(56%)
	Chair	18	(16%)
	Bare floor	15	(14%)
	Mat on the floor	10	(9%)
	Standing	5	(5%)
Feeding method, N = 110	Bowl	55	(50%)
	Bowl and spoon	20	(18%)
	Bowl, caregiver hands, and spoon	12	(11%)
	Bowl, infant hands, and spoon	6	(5%)
	Bowl and infant hands	6	(5%)
	Bowl and caregiver hands	5	(5%)
	Infant hands	2	(2%)
	Spoon	2	(2%)
	Caregiver hands	1	(1%)
	Cooking vessel	1	(1%)
Feeding utensil cleaned with soap and water before use, N = 109	No	96	(88%)
	Yes	13	(12%)
	Child fed directly from bowl without utensil	1	–
Feeding utensil appears clean, N = 109	Yes	107	(98%)
Liquids given to child during feeding, N = 110	Water	48	(44%)
	None	47	(43%)
	Tea with or without milk	10	(9%)
	Milk	5	(5%)
**Observed practices when storing remaining food**			
Hot food cooled, N = 82	Yes	69	(84%)
	No	13	(16%)
	Food was not hot at time of observation	28	–
Where hot food cooled, N = 86	Inside	48	(56%)
	Outside	38	(44%)
	Food was not hot at time of observation	24	–
Leftover infant food, N = 110	Yes	67	(61%)
Leftover infant food storage, N = 67	Sealed container	27	(40%)
	Fridge	14	(21%)
	Cooking vessel	11	(16%)
	Discarded	11	(16%)
	Loose or uncovered container	4	(6%)
	No food remained after feeding	43	–
**Household environmental conditions, N = 110**			
Animals present in food preparation area	Yes	12	(11%)
Animals present in child feeding area	Yes	11	(10%)
Flies visible in food preparation area	Yes	22	(20%)
Flies visible on food	Yes	16	(15%)
Garbage visible	Yes	26	(24%)

### Observed practices during food preparation

Most households prepared fresh food (69%), while 31% fed their child food that had been prepared prior to the structured observation. Among households that fed prepared foods, most reheated leftovers to boiling (65%). Fried eggs were served immediately and never stored, while salad was the only uncooked food. Of the 314 raw ingredients observed, most were stored inside the home (89%). Among ingredients for which storage was applicable, 90% were covered (n = 284). Over half of caregivers prepared food inside their home (57%) and on a table (70%). Of the 77 caregivers who prepared food on a table, less than 3% cleaned the food preparation surface with soap or detergent. Fewer than a quarter of caregivers washed their hands before food preparation (24%). Most caregivers did not add any liquids during cooking (56%), though some added milk or water (44%). Raw and cooked foods were typically separated during preparation (93%).

### Observed practices before child feeding

Few caregivers added any liquids after cooking (13%). Of these, 55% heated the liquid to boiling, while 45% did not. Only 15% of caregivers (or other persons responsible for feeding the child) and 4% of children washed their hands with soap and water before feeding. Most caregivers did not wash the child feeding utensil before use (88%), although most utensils appeared clean (98%).

### Observed practices during feeding the child

Caregivers were the main person feeding the child (87%), though some children were fed by someone other than the primary caregiver (7%) or jointly with another individual (e.g., siblings or an aunt) (5%). Most children were seated on their caregiver’s knee or lap during feeding (56%), followed by a chair (16%) or the floor (14%). Almost half of children did not consume liquids during feeding (43%), while a similar proportion drank water (44%). Bowls were the most common feeding vessel (50%), followed by a bowl and spoon (18%), and a combination of bowl, spoon, and caregiver hands (11%).

### Observed practices when storing and eating remaining food

Sixty-one percent of households stored infant food, while the remaining households (39%) did not have leftovers. Remaining infant food was typically stored in a sealed container (40%), in a fridge (21%), in a cooking vessel (16%), discarded (16%), or in a loose or uncovered container (6%). Most hot food was cooled before storing (84%) and typically cooled inside the household (56%).

### Household environmental conditions

Some households had animals present in the food preparation area (11%) and in the child feeding area (10%). Flies were visible in some food preparation areas (20%) and on children’s food (15%). Garbage was visible in approximately a quarter of households.

### Critical control points

Seven CCPs were identified using the HACCP approach, including handling, preparation, cooking, cooling, feeding, storing, and reheating. These CCPs represent points at which contamination could occur if an appropriate control measure is not implemented.

### Food flow diagram

**Fig 3:**
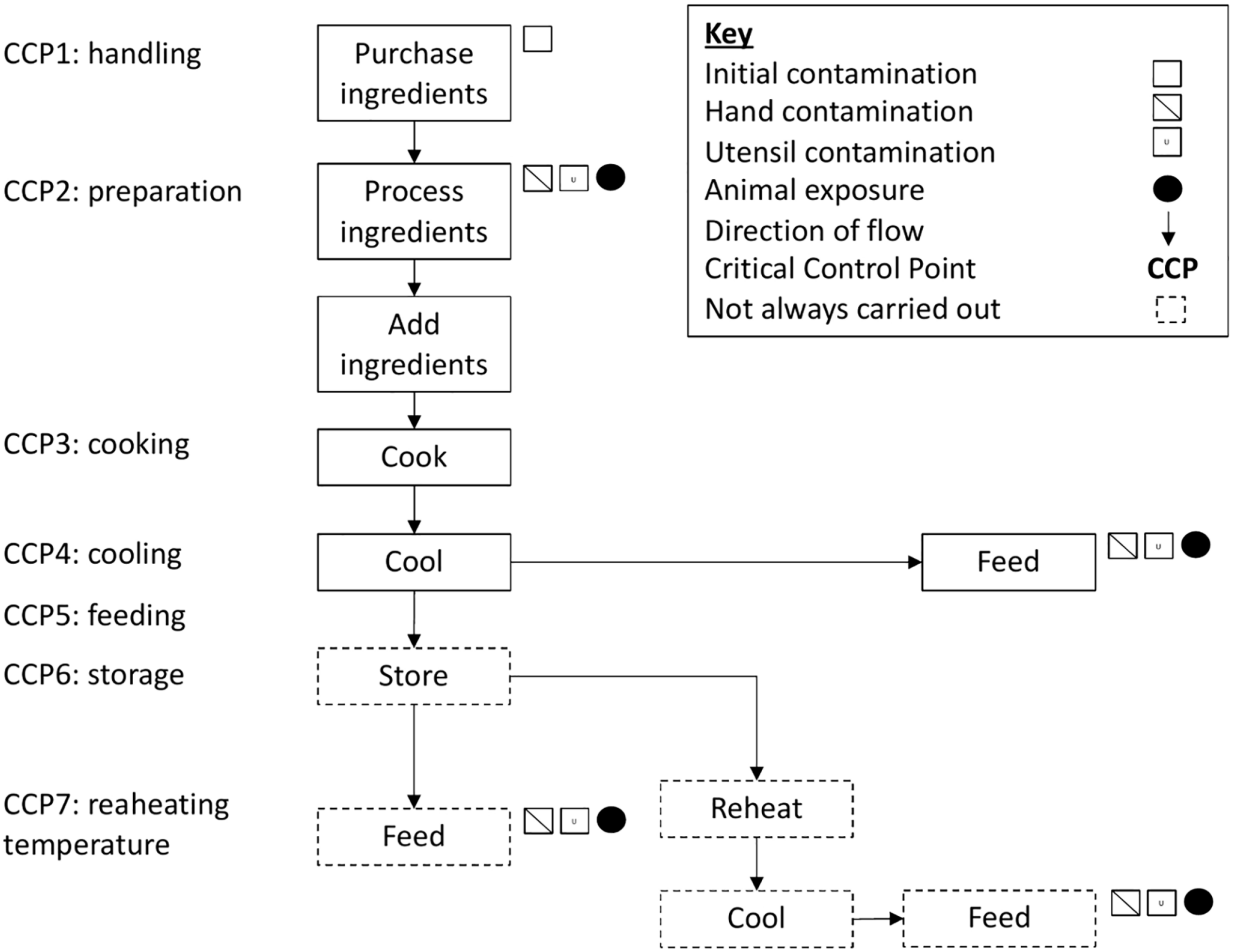
Food flow diagram for the preparation, feeding, and storage of all food types in households of children in Maputo, Mozambique.

Observation data were used to produce a food flow diagram for each food type, with CCPs indicated (S1 Fig–S8 Fig). The individual food flow diagrams were combined to produce an overall food flow diagram (Fig 3). CCPs were identified based on their potential role in pathogen destruction, survival, and/or propagation.

### Control measures

Control measures are suggested in Table 3 addressing all food categories and associated CCPs. CCPs for the specific food groups are outlined in the Supplemental Materials (S1 Fig – S8 Fig).

**Table 3:** Control measures and key behavioural actions for all food categories.

Control measure	Key behavioural actions	CCP
Handwashing with soap at key moments	Caregiver washing hands with soap and water before preparing food and feeding the child	• Handling
Cleaning with soap and water	Washing all utensils with a cleaning agent (e.g., dishwashing bar, dishwashing liquid, detergent powder) and water before use for food preparation and serving	• Handling • Preparation
Cooking and reheating food thoroughly	Cooking meals and reheating all stored foods until boiling before feeding the child	• Cooking • Reheating
Safe food storage	Cooling food before storing and storing food with lids and on a raised shelf or inside a cabinet to protect from flies, insects, and domestic animals	• Cooling • Storage
Hygienic child eating area	Ensuring the child feeding area is visibly clean from flies, insects, and animals	• Feeding

## Discussion

This study assessed food hygiene practices for the preparation, feeding, and storage of foods consumed by children aged 6–24 months in the Polana Caniço neighbourhood of Maputo, Mozambique. We applied the HACCP approach to 110 structured observations to identify potential practices that contribute to contamination of foods consumed by children aged 6–24 months. We identified eight categories of foods commonly consumed by the children enrolled in the study: cereals, cereals with vegetables, cereals with meat or fish, cereals with legumes, fried eggs, starchy vegetables, bread, and salad. Seven CCPs were identified through the HACCP approach, including food handling during preparation, cooking practices, child feeding, covered food storage, storage temperature and time, and reheating temperature and time. Control measures included caregiver handwashing with soap before preparation and feeding the child, washing utensils with a cleaning agent, cooking foods fresh or reheating prepared food to boiling, covering leftover food, and ensuring the child feeding area is clean and free from animal contact. While these CCPs are consistent with those reported in similar settings, this study contributes context-specific evidence on the frequency of food hygiene practices in an urban informal settlement in Mozambique, where detailed observational data remain limited.

These findings are consistent with previous HACCP studies in Mozambique and other settings, which identified cooking, food handling, and storage as CCPs [7,8,13–15]. Two studies also identified boiling milk and water before child consumption as a CCP [14,15]. In rural South Sudan, Wells et al. (2024) found animal contact with the food preparation and feeding area as a CCP [10], whereas Bick et al. (2020) did not report this in neighbourhoods similar to Polana Caniço in Maputo, Mozambique [7]. In Nepal, CCPs included unwashed hands before food handling, unclean feeding utensils, and inadequate reheating and storage of leftovers [14], which are consistent with our findings. Our results reinforce the persistence of unsafe food hygiene practices in this context rather than the identification of new CCPs.

Handwashing with soap and water before food preparation and child feeding was infrequent and comparable to previous studies [7,10]. In our study, less than a quarter of caregivers washed their hands before preparing food. Structural barriers may help explain these low handwashing rates. Only half of households had a handwashing facility and approximately one third had soap present at the facility at the time of observation. In addition, most households had access to a basic water source, although this does not reflect the reliability, quantity, or accessibility of water needed for handwashing. This study did not assess these specific aspects and therefore could not determine whether water availability influenced handwashing behaviour. This finding is consistent with previous studies showing that lack of a designated household handwashing facility is a barrier to handwashing with soap at key moments [11,20]. Hygiene promotion efforts should therefore prioritise access to handwashing facilities and availability of soap and water, alongside behaviour-centred health messaging. Future research should further examine how broader WASH conditions, including water access, influence handwashing practices to better inform food hygiene interventions.

Cleaning of feeding utensils and food preparation surfaces was rarely observed, consistent with other studies [7,10,14]. While cleaning of utensils occurred in only 12% of observations, almost all feeding utensils appeared clean. However, visual appearance of cleanliness does not necessarily indicate microbiological safety, as utensils can still be a potential route of transmission for faecal-oral infections if not stored properly and not cleaned before use [21]. Food preparation surfaces may also be contaminated with microorganisms if not properly cleaned and should be washed just prior to use to minimise the risk of foodborne disease [20].

Most food observed in this study was cooked to boiling, which is generally sufficient to reduce pathogen load, posing a minimal hazard to the child if consumed immediately after cooking. However, 35% of households that consumed pre-prepared food did not reheat it to boiling. Thorough cooking and reheating of food is critical for destroying pathogens [21], though it is difficult to set precise temperature thresholds in domestic environments as households usually do not have equipment to monitor temperature. This is particularly important when foods are consumed without reheating or when reheating temperatures are well below levels capable of destroying pathogens [21]. ‘Piping hot’, commonly used to define adequate cooking temperature, is a suitable alternative where temperature cannot be measured [21]. Nonetheless, future messaging can focus on boiling to destroy pathogens, as well as reheating to make the food warm and palatable.

Three quarters of households stored food at room temperature in either covered or uncovered containers, with only 13% of households storing food in a refrigerator. Storing food at room temperature favours the growth of microorganisms [21], as warmer conditions support microbial survival and proliferation [22]. Foods with high moisture content, such as cooked staples (e.g., rice and potatoes) and animal-source foods, provide favourable conditions for bacterial growth when stored at room temperature due to their high-water activity [22]. Such foods may also not always be reheated to sufficiently high temperatures before subsequent feedings, increasing the risk of foodborne illness [23]. In contrast, lower-moisture foods, such as dry cereals, are generally less susceptible to rapid bacterial growth due to their low water activity [24]. In addition, contamination levels increase when food is stored following repeated feedings during the day [25]. Few households owned a fridge. In the absence of refrigeration, leftover foods should be stored in clean, sealable containers for limited time periods. Preventing food contamination before and during storage is critical for ensuring that pathogens cannot multiply or be introduced prior to consumption [21].

Most children were fed sitting on caregiver’s lap, though some were fed while sitting directly on the floor, which may increase the risk of contamination. Children eating on the floor poses a public health risk as the child could inadvertently consume soil during feeding. Several studies have shown that soil is an important environmental transmission pathway of enteric pathogens [26–28]. This is especially relevant where children are in close contact with animals, as soil may contain animal faeces. Contact with animals during key moments, such as food preparation and child feeding, was observed in our study. Other studies also found that animals and the presence of animal faeces in the compound was associated with food contamination [9,29,30].

The identified CCPs and corrective measures are consistent with other HACCP studies [8,13–15] and can be used to develop food hygiene promotion interventions and target resources. These findings provide context-specific evidence to support the prioritisation of interventions in urban informal settlements in Maputo, Mozambique, where observational data on food hygiene practices remain limited. This localised evidence is important given that the frequency of unsafe food hygiene behaviours, as well as implementation constraints, may differ across settings included in other HACCP studies. In this setting, we recommend prioritising five key behaviours, including handwashing with soap at key moments, cleaning food preparation surfaces and utensils with soap and water, cooking and reheating foods until boiling, and safe food storage. Future interventions may also consider both infrastructural and behavioural determinants of handwashing with soap.

### Limitations

This study has several limitations. First, no food samples were collected to confirm the presence of microbial contamination in the food observed as part of the HACCP assessment. Future research should consider taking repeated food samples throughout the food preparation, feeding, and storage processes to better identify where contamination occurs and to quantify its magnitude. Second, as this is a cross-sectional study, it identifies potential hazards for food contamination but cannot establish causal relationships between risk factors and contamination. Third, the data are based on structured observations, which may have introduced reactivity due to the Hawthorne effect, especially given the relatively short observation period [31]. To minimise this, enumerators informed caregivers that the study focused on what foods the child eats, how they are prepared, and how feeding occurs, without explicitly stating that food hygiene practices were being observed. Data could also have been biased by enumerator reporting due to the observational nature of the study. Finally, the duration of food storage prior to observation and during the observation period was not measured, which may limit our ability to fully understand the relationship between storage practices and potential contamination risks. Future studies should measure storage duration to better characterise these risks.

## Conclusion

Our study highlights several hazards and potential control strategies that can reduce food contamination among foods consumed by children aged 6–24 months in Maputo, Mozambique. Future research may consider these hazards and critical control points to inform the design of interventions targeting key behaviours for infectious disease control at the household and community level in urban settings.

## Supporting information

S1 Checklist Inclusivity in global research questionnaire. (DOCX)

S1 Fig Food flow diagram for cereal-based foods. Food flow diagram for the preparation, feeding and storage of cereal-based food (e.g., porridge, rice, and xima), with the associated critical control points, among households of children between 6–24 months in Maputo, Mozambique.
(DOCX)

S2 Fig Food flow diagram for cereals with vegetables. Food flow diagram for the preparation, feeding and storage of cereals with vegetables, with the associated critical control points, among households of children between 6–24 months in Maputo, Mozambique.
(DOCX)

S3 Fig Food flow diagram for cereals with meat or fish. Food flow diagram for the preparation, feeding and storage of cereals with meat or fish, with the associated critical control points, among households of children between 6–24 months in Maputo, Mozambique.
(DOCX)

S4 Fig Food flow diagram for cereals with legumes. Food flow diagram for the preparation, feeding and storage of cereals with legumes (e.g., beans), with the associated critical control points, among households of children between 6–24 months in Maputo, Mozambique.
(DOCX)

S5 Fig Food flow diagram for fried eggs. Food flow diagram for the preparation, feeding and storage of fried egg, with the associated critical control points, among households of children between 6–24 months in Maputo, Mozambique.
(DOCX)

S6 Fig Food flow diagram for starch vegetables. Food flow diagram for the preparation, feeding and storage of starch vegetables, with the associated critical control points, among households of children between 6–24 months in Maputo, Mozambique.
(DOCX)

S7 Fig Food flow diagram for salad. Food flow diagram for the preparation, feeding and storage of salad, with the associated critical control points, among households of children between 6–24 months in Maputo, Mozambique.
(DOCX)

S8 Fig Food flow diagram for bread. Food flow diagram for the preparation, feeding and storage of bread, with the associated critical control points, among households of children between 6–24 months in Maputo, Mozambique.
(DOCX)
