# Supplementary material for: Hazard analysis and critical control points for foods consumed by children aged 6–24 months in Maputo, Mozambique
Source: PLOS Glob Public Health. 2026 Jul 7;6(7):e0005497. doi: 10.1371/journal.pgph.0005497 (PMC13340842; doi:10.1371/journal.pgph.0005497)
Supplement: S1 Checklist — (DOCX) [file pgph.0005497.s001.docx]

Inclusivity in global research

PLOS’ policy on inclusivity in global research aims to improve transparency in the reporting of research performed outside of researchers’ own country or community and ensures that PLOS publications reporting global research adhere to high standards for research ethics and authorship. Authors of relevant research articles may be asked to complete the questionnaire below, which outlines ethical, cultural, and scientific considerations specific to inclusivity in global research. This questionnaire may be requested when researchers have travelled to a different country to conduct research, if research uses samples collected in another country, research with Indigenous populations or their lands, or if research is on cultural artefacts. Researchers travelling to another country solely to use laboratory equipment will not normally be required to complete the questionnaire. However, the questionnaire can be requested at the journal’s discretion for any submission – if you have been requested to complete this questionnaire by the PLOS journal you submitted to, please do so.

Please complete the questionnaire below and include this as a Supporting Information file with your manuscript. Note that if your paper is accepted for publication, this checklist will be published with your article in the supporting information files. Please ensure that you reference the checklist in the main body of your manuscript. We suggest adding a subsection ‘Inclusivity in global research’ to your Methods section and adding the following sentence: “Additional information regarding the ethical, cultural, and scientific considerations specific to inclusivity in global research is included in the Supporting Information (S1 Checklist)”

The questions have been designed to be applicable to a wide range of study types, and there are subsections for both human subjects research and non-human subjects research. If any of the questions are not relevant to your research please mark them as “N/A” as appropriate.

**Ethical considerations, permits and authorship**

*This section is applicable to all research types.*

Provide details as to who granted permissions and/or consent for the study to take place in the Methods section of your manuscript. This should include the names of **all** ethics boards, governmental organizations, community leaders or other bodies that provided approval for the study. If individuals provided approval refer to these people by their role or title but do not list their name(s).

We have reported this in the methods section on page 5 of the manuscript:

Ethical approval was obtained from the London School of Hygiene and Tropical Medicine, Research Ethics Committee, London, UK (Ref: 17188) and National Committee on Bioethics for Health (CNBS) Comité Nacional de Bioética para a Saúde (CNBS) (Ref: 591/CNBS/19) in Mozambique. All study participants provided written informed consent (for both their and their child’s participation).

If there were any deviations from the study protocol after approval was obtained please provide details of these changes in the Methods section of your manuscript.

No deviation

Did this study involve local collaborators that are residents of the country where the research was conducted or members of the community studied? If you do not have any authors from said communities, please provide an explanation for this below.

Yes, eight co-authors of the 12 co-authors are nationals and residents of Mozambique where this research was conducted.

Everyone listed as an author should meet PLOS’ criteria for authorship and all individuals who meet these criteria should be included in the author byline, rather than the acknowledgements. For further information please see the journal’s Authorship Policy.

**Human subjects research (e.g. health research, medical research, cross-cultural psychology)**

Did you obtain written informed consent from a representative of the local community or region before the research took place? How did you establish who speaks for the community? Details of written informed consent obtained from study participants should be reported separately in the Methods section of your manuscript.

Written informed consent at the community level was obtained prior to the commencement of data collection. Community entry was facilitated through the Polana Caniço Health Research and Training Centre (CISPOC), National Institute of Health, Mozambique, which is the established local research authority in the study area. CISPOC engaged with local administrative and community leadership structures in Polana Caniço, including neighbourhood leaders and block-level representatives, who represent the local population. These structures were used to ensure appropriate community-level permission and to inform local stakeholders about the study objectives and procedures prior to household recruitment. Community leaders were identified based on CISPOC’s historic and ongoing work in Polana Caniço, where CISPOC is also based. Written informed consent from individual study participants is described separately in the Methods section of the manuscript.

How did members of the local community provide input on the aims of the research investigation, its methodology, and its anticipated outcome(s)?

The institution responsible for the study has a Community Advisory Board (CAB) composed of community members and local leaders. The CAB has been continuously involved throughout the study, including in the development of the research aims, discussions on methodological procedures, assessment of the study’s relevance to the local context, and review and interpretation of study findings.

The involvement of the Community Advisory Board aims to ensure that the study is conducted in an ethical and culturally appropriate manner, while remaining aligned with the priorities and needs of the communities involved.

When engaging with the local community, how did you ensure that the informed consent documents and other materials could be understood by local stakeholders?

Simple, clear, and culturally appropriate language was used to ensure that the local community adequately understood the informed consent forms. In the case where a participant could not read or write, a witness of their choice was identified to explain the study aim and procedures, as well as witness the thumbprinting of the informed consent form. In addition, field workers receive specific training on appropriate communication approaches for the communities involved in the study. Throughout the consent process, participants are encouraged to ask questions, clarify doubts, and request additional information to ensure that all aspects of the study were fully understood before participation.

Will the findings of the research be made available in an understandable format to stakeholders in the community where the study was conducted (e.g. via a presentation, summary report, copies of publications, etc.)? Please provide details of how this will be achieved.

The findings from this research were presented at the XVIII Jornadas Nacionais de Saúde (18th National Health Conference), hosted by the National Institute of Health, from 1–3 October 2025 in Maputo, Mozambique.

the national INS conference in October 2025 in Maputo, Mozambique.

**Non-human subjects research using specimens/ animals collected as part of the study, or those housed in archival collections. Examples include archaeology, paleontology, botany and zoology.**

Did the permission you obtained from a local authority to perform the study include an agreement on access to outputs and benefit sharing? This may include procedures to enable fair distribution of the benefits and resources arising from the research performed. Please include any details of Prior Informed Consent and Benefit Sharing Agreements obtained. These may be required by field-specific regulations, for example the Convention on Biological Diversity (CBD) and the associated Nagoya Protocol.

N/A

If the material used in your study was imported, please A) provide the year it was imported and B) indicate whether permits were obtained to import/export the materials used, C) provide details of any permits obtained. If this information is not available, please indicate this.

N/A

If you used archival specimens, please state how the material used in your study was acquired by the institute it is held in and provide details of any permits obtained for the original excavations/ sample collection. If this information is not available, please indicate this.

N/A

How was the potential cultural significance of the materials collected in your study to local communities considered in your research design? Were Indigenous peoples and/or local researchers and institutions involved with archaeological excavations / collection of specimens? If so, please provide a description of their involvement.

N/A

If your manuscript includes photographs of human remains please indicate whether authors obtained permission from descendants or affiliated cultural communities to do so.

N/A
