# Supplementary material for: Hazard analysis and critical control points for foods consumed by children aged 6–24 months in Maputo, Mozambique
Source: PLOS Glob Public Health. 2026 Jul 7;6(7):e0005497. doi: 10.1371/journal.pgph.0005497 (PMC13340842; doi:10.1371/journal.pgph.0005497)
Supplement: S3 Fig — Food flow diagram for the preparation, feeding and storage of cereals with meat or fish, with the associated critical control points, among households of children between 6–24 months in Maputo, Mozambique. (DOCX) [file pgph.0005497.s004.docx]

**Supporting Information**

**S3 Fig. Food flow diagram for cereals with meat or fish.** Food flow diagram for the preparation, feeding and storage of cereals with meat or fish, with the associated critical control points, among households of children between 6–24 months in Maputo, Mozambique.
