# Supplementary material for: Hazard analysis and critical control points for foods consumed by children aged 6–24 months in Maputo, Mozambique
Source: PLOS Glob Public Health. 2026 Jul 7;6(7):e0005497. doi: 10.1371/journal.pgph.0005497 (PMC13340842; doi:10.1371/journal.pgph.0005497)
Supplement: S5 Fig — Food flow diagram for the preparation, feeding and storage of fried egg, with the associated critical control points, among households of children between 6–24 months in Maputo, Mozambique. (DOCX) [file pgph.0005497.s006.docx]

**Supporting Information**

**S5 Fig. Food flow diagram for fried eggs.** Food flow diagram for the preparation, feeding and storage of fried egg, with the associated critical control points, among households of children between 6–24 months in Maputo, Mozambique.
